# Procyanidins Mitigate Osteoarthritis Pathogenesis by, at Least in Part, Suppressing Vascular Endothelial Growth Factor Signaling

**DOI:** 10.3390/ijms17122065

**Published:** 2016-12-09

**Authors:** Angela Wang, Daniel J. Leong, Zhiyong He, Lin Xu, Lidi Liu, Sun Jin Kim, David M. Hirsh, John A. Hardin, Neil J. Cobelli, Hui B. Sun

**Affiliations:** 1 Departments of Orthopedic Surgery, Albert Einstein College of Medicine, Bronx, NY 10461, USA; angela.wang@einstein.yu.edu (A.W.); daniel.leong@einstein.yu.edu (D.J.L.); zhiyong.he@einstein.yu.edu (Z.H.); lin.xu@einstein.yu.edu (L.X.); lidi.liu@einstein.yu.edu (L.L.); sukim@montefiore.org (S.J.K.); dhirsh@montefiore.org (D.M.H.); jhardin@montefiore.org (J.A.H.); ncobelli@montefiore.org (N.J.C.); 2 Departments of Radiation Oncology, Albert Einstein College of Medicine, Bronx, NY 10461, USA

**Keywords:** procyanidins, post-traumatic osteoarthritis, chondroprotection, osteoarthritis, VEGF, nutraceuticals, pine bark extract

## Abstract

Procyanidins are a family of plant metabolites that have been suggested to mitigate osteoarthritis pathogenesis in mice. However, the underlying mechanism is largely unknown. This study aimed to determine whether procyanidins mitigate traumatic injury-induced osteoarthritis (OA) disease progression, and whether procyanidins exert a chondroprotective effect by, at least in part, suppressing vascular endothelial growth factor signaling. Procyanidins (extracts from pine bark), orally administered to mice subjected to surgery for destabilization of the medial meniscus, significantly slowed OA disease progression. Real-time polymerase chain reaction revealed that procyanidin treatment reduced expression of vascular endothelial growth factor and effectors in OA pathogenesis that are regulated by vascular endothelial growth factor. Procyanidin-suppressed vascular endothelial growth factor expression was correlated with reduced phosphorylation of vascular endothelial growth factor receptor 2 in human OA primary chondrocytes. Moreover, components of procyanidins, procyanidin B2 and procyanidin B3 exerted effects similar to those of total procyanidins in mitigating the OA-related gene expression profile in the primary culture of human OA chondrocytes in the presence of vascular endothelial growth factor. Together, these findings suggest procyanidins mitigate OA pathogenesis, which is mediated, at least in part, by suppressing vascular endothelial growth factor signaling.

## 1. Introduction

Osteoarthritis (OA) is a degenerative joint disease affecting more than 27 million Americans and is a leading cause of adult disability [1]. It is characterized by progressive degradation and eventual loss of articular cartilage [2,3]. The risk of developing OA is associated with increased age and overuse or injury to the articular joint [4,5,6,7,8,9,10]. Despite the increasing prevalence of OA, an effective and safe treatment for OA has not yet been established [11]. Pharmacologic treatments for symptomatic relief of OA, such as nonsteroidal anti-inflammatory drugs (NSAIDs), show negligible efficacy in disease modification [12], and may cause gastrointestinal, renal and cardiovascular side effects [12]. There is a great need for new OA interventions that can slow or stop progression of the structural damage associated with OA while reducing symptoms. Because OA is a disease that often extends over decades, such agents must be safe to permit their use over extended time periods [13].

Nutraceuticals have shown efficacy in the prevention and treatment of osteoarthritis (reviewed in [14,15]). For example, reduction of symptoms have been documented with the use of curcumin [16], extract of green tea [17], and extra virgin olive oil [18]. As a part of this group of generally recognized as safe (GRAS) plant-based products, procyanidins are a family of plant metabolites found in fruits, vegetables, nuts, seeds, flowers, and bark that have been shown to have beneficial biological activities, including anti-oxidative [19,20,21], anti-cancer [22,23,24], and vasorelaxing effects [25,26,27]. The composition of procyanidins include catechin, epicatechin, procyanidins B1–B5 and procyanidin C1 [28]. The therapeutic or potentially therapeutic effects of these individual compounds have been explored (Table 1).

In vitro studies have shown procyanidins suppress interleukin (IL)-1β-induced increases in reactive oxygen species (ROS) production in human chondrocytes [29]. In animal studies, grape seed extract, rich with procyanidins, reduce ROS activity and expression of matrix metalloproteinases (MMPs) in the articular cartilage of monosodium iodoacetate-induced OA rats [30], and procyanidins extracted from grape seeds ameliorate collagen-induced arthritis (CIA) in mice through regulation of the toll-like receptor 4 (TLR4)/myeloid differentiation factor 88 (MyD88)/nuclear factor-κB (NF-κB) signaling pathway [31]. Notably, daily oral administration of procyanidin B3, one of the components of procyanidins, was shown to mitigate OA pathogenesis in mice subject to medial collateral ligament transection and medial meniscectomy by, at least in part, suppressing induced nitric oxide synthase (iNOS) [32]. However, whether procyanidins, procyanidin B2 and procyanidin B3, or other components of procyanidins exert chondroprotective effects via other OA-relevant pathways is unknown.

Vascular endothelial growth factor (VEGF) is a signal protein produced by cells that stimulates vasculogenesis and angiogenesis [52]. VEGF plays a critical role in enhancing new blood vessel formation during embryonic development and tissue injury repair. However, it may contribute to disease when it is overexpressed [53]. VEGF has been suggested to play a key role in OA pathogenesis [54]. VEGF is expressed during articular cartilage growth [55]. While the expression of VEGF is largely quiescent during maturity in adult non-OA cartilage, its expression is elevated in OA cartilage [56,57,58,59]. Furthermore, intra-articular injections of VEGF into the mouse knee joint [60] and the temporomandibular joint [61] induce OA, and intra-articular injections of VEGF-specific antibody bevacizumab mitigates OA progression in OA rabbits [62]. VEGF has also been demonstrated to regulate several pathways in OA pathogenesis, such as those involved in oxidative stress and catabolism [63]. For example, VEGF mediates OA progression by increasing expression of pro-inflammatory cytokines, including IL-1β, IL-6, and tumor necrosis factor α (TNF-α), proteases such as matrix metalloproteinases (MMPs, e.g., MMP-1, MMP-3, MMP-9, MMP-13), and A disintegrin and metalloproteinase with thrombospondin motifs 5 (ADAMTS5), and decreasing expression of cartilage matrix proteins type II collagen and aggrecan (reviewed in [54]). Of interest, procyanidins have been shown to inhibit expression of VEGF in cancer cells [64,65] and endothelial cells [66]. However, whether procyanidins exert an effect on VEGF and its mediated signaling in OA pathogenesis is unknown.

The aim of this study was to test the hypothesis that procyanidins prevent and/or mitigate OA pathogenesis by, at least in part, suppressing VEGF. We tested this hypothesis in vivo using destabilization of the medial meniscus (DMM)-induced OA mice, and in vitro using human primary OA chondrocytes.

## 2. Results

### 2.1. Procyanidins Slowed Destabilization of the Medial Meniscus (DMM)-Induced Osteoarthritis (OA) Disease Progression in Mice

**Figure 1:**
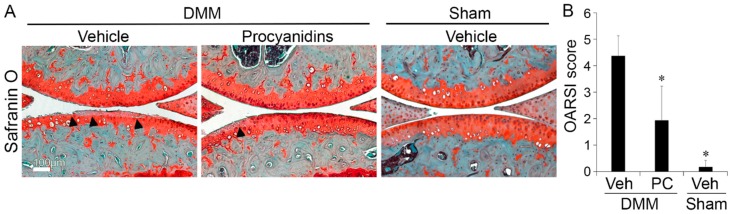
Oral administration of procyanidins slowed destabilization of the medial meniscus (DMM)-induced OA disease progression in mice. Mice subjected to DMM were treated daily with procyanidins (PC) or vehicle (Veh) by oral gavage. The articular cartilage of the DMM-wounded knee in mice treated with procyanidins exhibited a reduced loss of cartilage as visualized by Safranin-O staining (**A**) and lower Osteoarthritis Research Society International (OARSI) scores (**B**) at 8 weeks following surgery compared to the articular cartilage of the DMM-wounded knee in mice treated with vehicle. Black arrows denote representative areas of cartilage degradation, such as proteoglycan loss, cartilage fibrillation and/or erosion. Regions of red indicate staining of proteoglycans in the articular cartilage, meniscus, or growth plate, by Safranin-O. The regions of light blue-green indicate subchondral bone. Scale bar = 100 µm. * *p* < 0.05 by *t*-test; *n* = 4/group.

The articular cartilage in the knee joint of mice subjected to DMM and treated daily for eight weeks with phosphate-buffered saline (PBS), the vehicle control, exhibited a moderate-OA pathological change characterized by loss of proteoglycans in the articular cartilage extracellular matrix (ECM), as revealed by loss of Safranin-O staining, increased cartilage fibrillation, and increased cartilage erosion (Figure 1A), quantified by Osteoarthritis Research Society International (OARSI) score of 4.4 ± 0.7. (Figure 1B). In contrast, the cartilage in the knee joint of mice subjected to DMM and treated with procyanidins (extracts from pine bark) via daily oral delivery exhibited reduced proteoglycans loss, cartilage fibrillation and erosion (Figure 1A) with a significantly lower OARSI score (1.9 ± 1.3), compared to that of mice treated with vehicle controls (*p* < 0.05, Figure 1B). Sham animals treated with vehicle control exhibited mild cartilage degradation (Figure 1A), with an OARSI score of 0.18 ± 0.24 (Figure 1B).

### 2.2. Procyanidins Reduced Levels of Cleaved Type II Collagen and Cleaved Aggrecan in the Articular Cartilage of Mice Subjected to DMM Surgery

**Figure 2:**
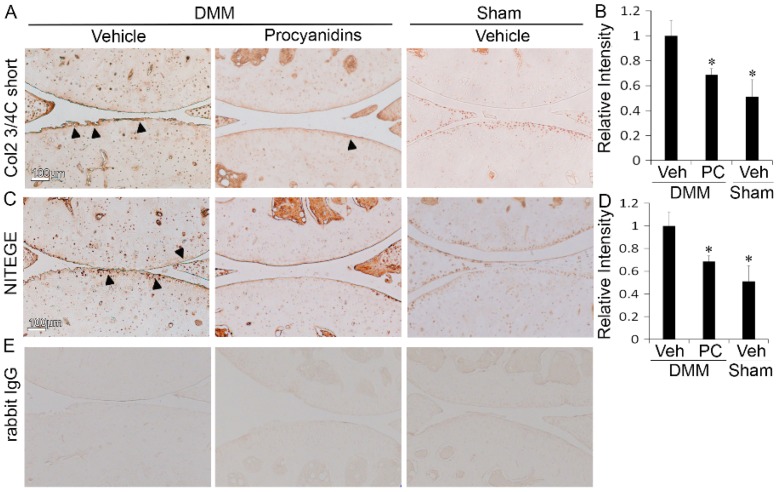
Oral administration of procyanidins reduced matrix degradation of articular cartilage in mice subjected to DMM surgery. Immunohistochemical staining of type II collagen cleavage epitope (Col2 3/4C short) (**A**) and cleaved aggrecan (NITEGE) (**C**); The relative staining intensities of Col2 3/4C short (**B**); and NITEGE (**D**) of the articular cartilage matrix in mice subjected to DMM and treated with procyanidins (PC) were significantly reduced compared to that in mice subjected to DMM and treated with vehicle (Veh); (**E**) representative immunostaining of tissue sections with isotype control (rabbit immunoglobulin G, IgG). Scale bar = 100 µm. * *p* < 0.05 by one-way ANOVA with Tukey post-hoc test; *n* = 4/group. Arrows denote representative regions with positive immunostaining of type II collagen cleavage epitope (Col2 3/4C short) (**A**) and cleaved aggrecan (NITEGE) (**C**).

To further evaluate the therapeutic effect of procyanidins in mitigating OA pathogenesis, we compared the proteolytic cleavage status of the main components of the cartilage extracellular matrix, type II collagen and aggrecan, in procyanidin-treated versus vehicle-treated mice subjected to DMM surgery. Oral administration of procyanidins reduced the levels of the type II collagen cleavage epitope (Col2 3/4C short) in mice subjected to DMM compared to that in the vehicle control (Figure 2A). The immunostaining intensity for cleaved type II collagen was reduced to 0.69-fold in procyanidin-treated animals compared to that of vehicle-treated controls (*p* < 0.05, Figure 2B). The immunostaining intensity was 0.51-fold in vehicle treated mice subjected to sham surgery compared to that of vehicle treated mice subjected to DMM surgery. Similarly, immunostaining showed that oral administration of procyanidins reduced the levels of cleaved aggrecan (NITEGE) in mice subjected to DMM compared to those of mice subjected to DMM and treated with vehicle (Figure 2D). At 8 weeks after DMM, the immunostaining intensity of cleaved aggrecan in procyanidin-treated mice subjected to DMM was reduced to 0.64-fold compared to that of vehicle treated mice subjected to DMM (*p* < 0.05, Figure 2B). This immunostaining intensity was 0.51-fold in vehicle treated mice subjected to sham surgery compared to that of vehicle treated mice subjected to DMM surgery.

### 2.3. Procyanidins Reduced Protein Levels of Matrix Metalloproteinase 13 (MMP-13) and a Disintegrin and Metalloproteinase with Thrombospondin Motifs 5 (ADAMTS5) in Cartilage of Mice Subjected to DMM Surgery

**Figure 3:**
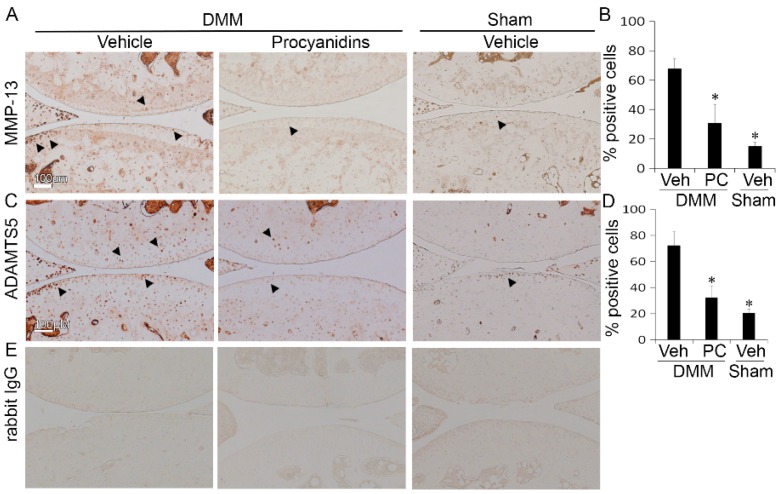
Oral administration of procyanidins reduced protein levels of matrix metalloproteinase (MMP)-13 and a disintegrin and metalloproteinase with thrombospondin motifs 5 (ADAMTS5) in articular cartilage of mice subjected to DMM surgery. Immunohistochemical staining of MMP-13 (**A**); and of ADAMTS5 (**C**) and percentage of positive cells (**B**,**D**) in the articular cartilage of mice subjected to DMM and treated with procyanidins (PC) was significantly reduced compared to that in mice subjected to DMM and treated with vehicle (Veh); (**E**) representative staining of tissue sections with isotype control (rabbit immunoglobulin G, IgG). Scale bar = 100 µm. * *p* < 0.05 by one-way ANOVA with Tukey post-hoc test; *n* = 4/group. Arrows indicate representative chondrocytes with positive immunostaining for MMP-13 (**A**) or ADAMTS5 (**C**).

To understand the mechanism of action underlying the cartilage protection that procyanidins exert, we examined the effect of procyanidins on the expression of two proteolytic enzymes MMP-13 and ADAMTS5, which are primarily responsible for cleavage of type II collagen and aggrecan [2,67], respectively. At 8 weeks following DMM, the percentage of MMP-13 positive cells was reduced from 68% in the cartilage of vehicle-treated mice to 31% in the cartilage of procyanidin-treated mice (*p* < 0.05, Figure 3B). In comparison, at 8 weeks following sham surgery, the percentage of MMP-13 positive cells was 15% in the articular cartilage of vehicle treated mice. Similarly, procyanidins reduced the percentage of ADAMTS5 positive cells from 72% in the cartilage of vehicle-treated to 32% in the cartilage of procyanidin-treated mice subjected to DMM surgery (*p* < 0.05, Figure 3D). The percentage of ADAMTS5 positive cells was 20% in the articular cartilage of vehicle treated mice subjected to sham surgery.

### 2.4. Procyanidins Suppressed Expression of Vascular Endothelial Growth Factor (VEGF) and Genes that May Be Regulated by VEGF and Involved in OA Pathogenesis in Cartilage of Mice with DMM-Induced OA

**Figure 4:**
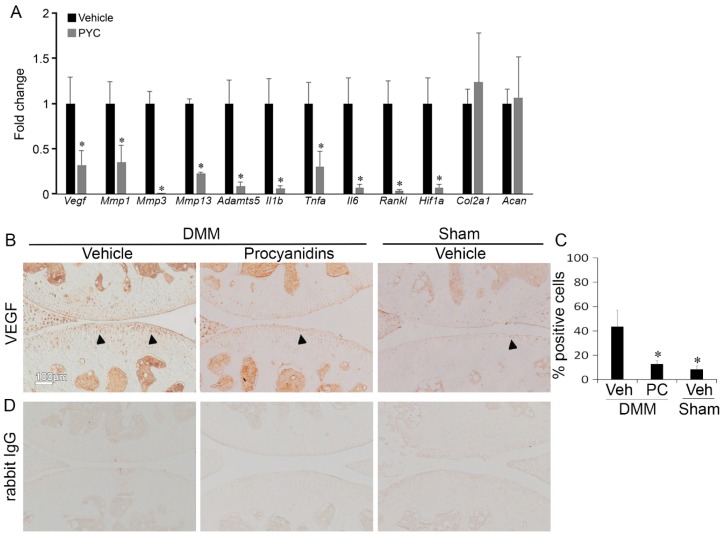
Oral administration of procyanidins suppressed expression of vascular endothelial growth factor (VEGF) and genes that may be regulated by VEGF and are involved in osteoarthritis (OA) pathogenesis in articular cartilage of mice subjected to DMM surgery. (**A**) Relative mRNA levels of *Vegf*,* Mmp1*,* Mmp3*,* Mmp13*,* Adamts5*, interleukin *(Il)1b* tumor necrosis factor α (*Tnfa*), *Il6*, receptor activator of nuclear factor kappa-B ligand (*Rankl*), and hypoxia-inducible factor (*Hif)1a* in articular cartilage of mice subjected to DMM treated with procyanidins were significantly reduced compared to that in mice subjected to DMM and treated with vehicle; (**B**,**C**) immunohistochemistry and quantification of chondrocytes expressing VEGF (* *p* < 0.05, *t*-test, *n* = 4/group); (**D**) representative staining of tissue sections with isotype control (rabbit immunoglobulin G (IgG)). * *p* < 0.05 by one-way ANOVA with Tukey post-hoc test; *n* = 4/group. Arrows indicate representative chondrocytes with positive staining for VEGF.

To identify the potential role of VEGF as a treatment pathway target of procyanidins in OA pathogenesis, we analyzed the gene expression of *VEGF* and array of genes that may be regulated by VEGF and involved in OA pathogenesis using the DMM-induced OA mouse model. Procyanidins significantly inhibited expression of *VEGF* and *VEGF* activator hypoxia-inducible factor 1-α (*Hif1*-α), and pathway associated mediators such as receptor activator of nuclear factor κ-B ligand (*Rankl*), pro-inflammatory cytokines *Il1b*, *Tnfa*, and *Il6*, proteolytic enzymes matrix metalloproteinases *Mmp1*, *Mmp3*, and *Mmp13* and aggrecanase *Adamts5* in the articular cartilage of mice with DMM-induced OA (Figure 4A). Expressions of type II collagen (*Col2a1*) and aggrecan (*Acan*) were not significantly affected by procyanidin treatment (Figure 4A). Procyanidin treatment also significantly reduced the number of chondrocytes immunostaining positive for VEGF in mice subjected to DMM surgery, as revealed by immunohistochemistry assay with 43% of cells stained positive in the cartilage of vehicle-treated compared to 12% in that of procyanidin-treated mice subjected to DMM. The percentage of VEGF positive cells was 8% in the articular cartilage of vehicle treated mice subjected to sham surgery (*p* < 0.05, Figure 4B).

### 2.5. Procyanidins Reduced Expression of VEGF and Phosphorylatison of VEGF Receptor in Primary Human OA Chondrocytes

**Figure 5:**
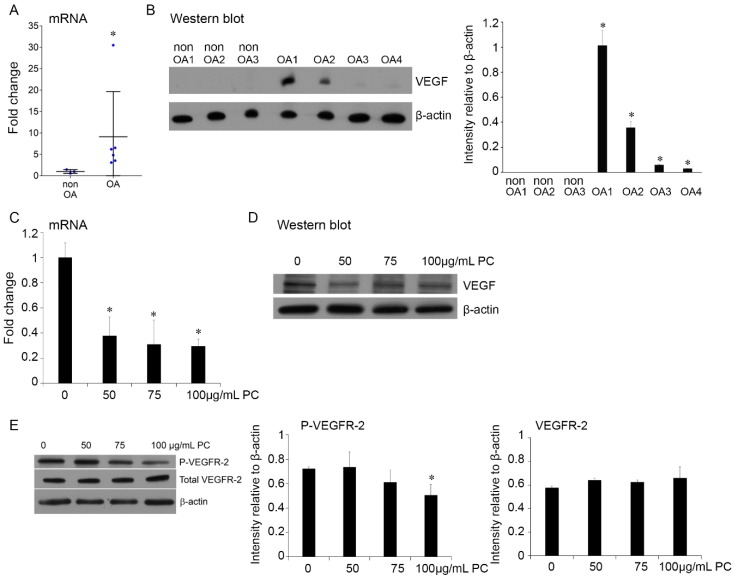
Procyanidins inhibited expression of VEGF and phosphorylation of VEGF receptor-2 in primary human OA chondrocytes. VEGF mRNA expression (**A**) and protein level (**B**) were significantly elevated in primary human OA chondrocytes compared to non-OA chondrocytes. Procyanidins (PC) inhibited *VEGF* mRNA expression (**C**) and protein expression (**D**) and phosphorylation of vascular endothelial growth factor receptor 2 (P-VEGFR-2) (**E**) in cultured primary human chondrocytes in the presence of human recombinant IL-1β (10 ng/mL). Western blot and quantifications of three representative Western blots are shown. * *p* < 0.05 vs. controls, one-way ANOVA with Tukey post hoc test.

To determine the potential of VEGF and its mediated signaling pathway as a target of procyanidins and the relevance to human OA disease treatment, we examined the effect of procyanidins on VEGF signaling, primarily focusing on the effect on phosphorylation of VEGF receptor, a key molecular event in the activation of the VEGF signaling pathway [68] in human primary chondrocytes. To this end, we first compared VEGF expression in primary chondrocytes derived from the articular cartilage of OA patients and primary chondrocytes from non-OA individuals. *VEGF* mRNA expression was significantly increased in chondrocytes derived from OA samples (Figure 5A). Consistent with previous studies [56,57,58,59], this study found that VEGF protein was undetectable in all three samples of non-OA chondrocytes. In contrast, the protein was detectable in all four samples of primary chondrocytes derived from OA individuals, and the protein level in two of the four cases was substantially increased as quantified by intensity relative to β-actin (Figure 5B). VEGF expression at the mRNA (Figure 5C) and protein levels (Figure 5D) were significantly reduced in cultured primary human OA chondrocytes treated with procyanidins at the concentration of 50, 75, and 100 µg/mL, with no significant difference within this dosage range. The procyanidin treatment at 100 µg/mL dose also significantly suppressed vascular endothelial growth factor receptor 2 (VEGFR-2) at phosphorylation level, which is critical in mediating a majority of the cellular responses to VEGF [68], but does not affect the total level of VEGFR-2 expression (Figure 5E).

### 2.6. Procyanidins B2 and B3 in Procyanidins Were Primarily Responsible for Reduction in VEGF Signaling

**Figure 6:**
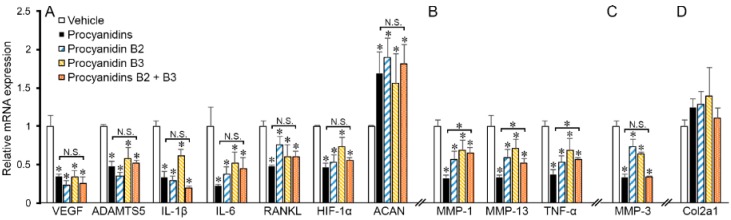
The effects of procyanidin B2 and B3 on expression of VEGF and genes that may be regulated by VEGF and involved in OA pathogenesis compared to those of procyanidins in primary human OA chondrocytes in the presence of human recombinant VEGF. mRNA expression of (**A**) *VEGF, ADAMTS5, IL-1β, IL-6, RANKL, HIF-1α* and aggrecan (*ACAN*); (**B**) *MMP-1, MMP-13, TNF-α*; (**C**) *MMP-3*; and (**D**) *Col2a1* in human primary OA chondrocytes treated with procyanidins (50 µg/mL), procyanidin B2 (1 µg/mL), procyanidin B3 (1.25 µg/mL) and procyanidins B2 + B3 (1 and 1.25 µg/mL respectively) in the presence of human recombinant VEGF (10 ng/mL). (* *p* < 0.05 vs. vehicle control or indicated comparison, one-way ANOVA with Tukey post-hoc test; N.S.: not significant, *p* > 0.05; *n *= 3/group).

Procyanidins are a combination of bioflavonoids and phenolic acids [69]. To identify the active ingredients of procyanidins that are responsible for its OA mitigating activity, we examined the effect of procyanidin B2 (B2) and procyanidin B3 (B3) on expression of VEGF and genes that may be regulated by VEGF and involved in OA pathogenesis in human primary OA chondrocytes in the presence of human recombinant VEGF (10 ng/mL). As shown in Figure 6, the total components of procyanidins, B2 and B3 individually, and B2 and B3 in combination, significantly modulated expression of all tested genes, except Col2a1. Treatment with B2 and B3 individually showed a similar effect to that of total components of procyanidins, and that of B2 and B3 in combination, in significantly reducing expression of *VEGF*,* ADAMTS5*,* IL-1β*, *IL-6*, *RANKL* and *HIF-1α*, and upregulated expression of aggrecan (*ACAN*; Figure 6A). B2, B3, and B2 and B3 in combination also significantly inhibited *MMP-1*, *MMP-13*, and *TNF-α*, but to a lesser degree compared to the effect of the total components of procyanidins (Figure 6B). Of note, procyanidin B2 and procyanidin B3 showed no addictive or synergistic effect, except in the case of suppressing *MMP-3*, where B2 and B3 in combination had an additive effect compared to that of B2 or B3 individually (Figure 6C).

## 3. Discussion

In this study, we show procyanidins mitigate OA pathogenesis in a DMM-induced posttraumatic OA mouse model. Treatment with procyanidins protected the integrity of the major cartilage matrix proteins type II collagen and aggrecan. Consistent with previous reports, we showed that procyanidin treatment reduced expression of MMP-13 [30], the most potent enzyme in cleaving type II collagen [3]. We also found that, for the first time based on our best knowledge, procyanidins suppressed expression of aggrecanases, in particular ADAMTS5, in chondrocytes both in vivo (Figure 3 and Figure 4) and in vitro (Figure 6). These data suggest that treatment with procyanidins improves the integrity of the articular cartilage by preserving both collagen and aggrecan components in DMM-induced OA in mice, and that the chondroprotective effects exerted by procyanidins are ultimately mediated by the action of suppressing MMP-13 and ADAMTS5. Of note, immunohistochemistry staining demonstrated procyanidin treatment reduced degradation of the articular cartilage matrix, such as cleavage of type II collagen and cleavage of aggrecan (Figure 2), while real time polymerase chain reaction (PCR) revealed no significant changes in mRNA expression of type II collagen and aggrecan in the cartilage of DMM mice treated with procyanidins (Figure 4). Together, these data suggest that procyanidins mitigate cartilage degradation mainly by protecting the cartilage extracellular matrix from degradation, rather than through anabolic means, such as by increasing expression of matrix proteins. While the targeted array of the gene expression analysis in vivo (Figure 4), and in vitro using human primary chondrocytes (Figure 6) is consistent, there is a discrepancy with the aggrecan mRNA results. This may be due to the variation between human and mouse models, and the experimental design, which will be validated in future studies.

**Figure 7:**
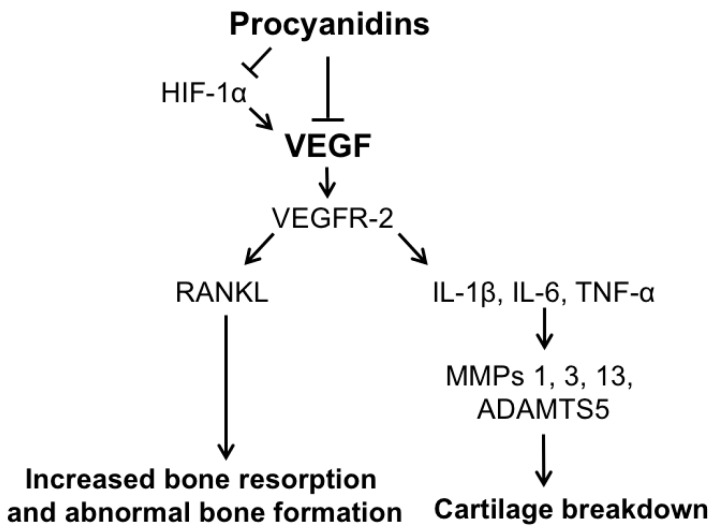
Hypothesized mechanism of procyanidin-mediated mitigation of OA pathogenesis. Procyanidins suppress expression of VEGF-mediated signaling by suppressing expression of VEGF and phosphorylation of VEGFR-2, and/or indirectly by reducing expression of HIF-1α. Suppression of VEGF-mediated signaling downregulates expression of VEGF and its signaling pathway associated mediators, including pro-inflammatory cytokines IL-1β, IL-6, and TNF-α, and proteolytic enzymes MMP-1, -3, -13, and ADAMTS5, which cleave components of the cartilage extracellular matrix. Procyanidins also suppress VEGF-induced RANKL, preventing increased bone resorption and abnormal bone formation.

While previous studies showed that procyanidins exert a chondroprotective effect in part by suppressing oxidative stress-induced factors [29,30,32], we revealed that procyanidins inhibit VEGF-induced signaling in the articular cartilage of DMM-induced OA mice. VEGF signaling plays a significant role in OA progression, including enhancing expression of pro-inflammatory cytokines and catabolic mediators in chondrocytes [54,70]. In OA, VEGF has also been linked to vascular invasion in the normally avascular cartilage, recruiting osteoblasts that lay down new bone matrix and osteoclasts that degrade the cartilage matrix [54]. VEGF has been associated with stimulation of RANKL, which aids in osteoclastogenesis in the subchondral bone and in turn secretes more VEGF into the articular cartilage to stimulate proteolytic enzymes and pro-inflammatory cytokines [61]. In this study, we show procyanidins reduced the expression of VEGF and VEGF activator hypoxia-inducible factor (Hif)1-α [71], and inhibited phosphorylation of VEGFR-2, which plays a major role in mediating the effects of VEGF [72]. Oral administration of procyanidins in mice subjected to DMM (Figure 4) and in human primary OA chondrocytes in the presence of VEGF (Figure 6) led to a significant reduction in expression of mediators downstream of VEGF, including *MMP-1*, *MMP-3*, *MMP-13*, *ADAMTS5*, *IL-1β*, *TNF-α*, *IL-6*, and* RANKL*. Together, these findings suggest that procyanidins mitigate OA pathogenesis, which is mediated, at least in part, by suppressing expression of VEGF and its signaling network (Figure 7). Elevated levels of VEGF and its pathways in osteoarthritic cartilage have been reported in the literature [56,57,58]. Interestingly, our data indicate that while VEGF was elevated in all four OA samples examined, compared to that in non-OA chondrocytes, the levels of VEGF protein expression varied significantly (Figure 5A,B). This finding suggests that the involvement and contribution of VEGF in OA disease pathogenesis may vary among OA patients. Of note, genetic variation adds to the complexity of OA disease progression [73], and single-nucleotide polymorphisms in the *VEGF* gene have been associated with OA development [74]. As procyanidins mitigate OA pathogenesis, at least in part through targeting VEGF signaling, procyanidins may exert a more profound therapeutic effect in OA patients with higher levels of VEGF.

The feasibility of treating OA by targeting VEGF has been previously demonstrated through the use of an anti-VEGF antibody in rabbits with OA [62]. However, adverse effects have been observed with anti-VEGF treatments, including vascular disturbances and regression of blood vessels [75], thus limiting their use chronically. Procyanidins are a combination of bioflavonoids and phenolic acids. In pine bark extract, 40.9% of procyanidins are found in a dimeric form [76], namely B procyanidins, of which B3 has shown efficacy in surgically-induced OA mouse model [32]. Additionally, tri- and tetramers have also shown inhibiting effects on VEGF signaling in endothelial cells in vitro and on tumor-induced blood vessel formation in vivo [77]. Indeed, the effects of procyanidins have been demonstrated to be greater than those of its isolated fractions [76,78,79]. Supported by other observations [31,32] and clinical data that have shown that procyanidins from pine bark extract reduce OA pain and inflammation [80,81], our study further suggests that, as generally recognized as safe (GRAS) biologically-active compounds, procyanidins and their active ingredients including procyanidin B2 and procyanidin B3, exert mitigating effects on OA pathogenesis. In particular, our study shows that procyanidins B2 and B3 can largely replicate the mitigating effects of procyanidins in VEGF-mediated OA pathogenesis, highly indicating that these compounds are the active ingredients in procyanidins in OA disease modification.

Our current study focused on the potential efficacies of procyanidins in OA disease initiation, and we have provided clear evidence that procyanidins mitigate the initiation of OA. We showed oral administration of procyanidins significantly slowed OA disease progression, suggesting a great potential for the clinical application in preventing or delaying OA disease initiation in relevant joint injuries patients who have a significantly higher frequency for development of posttraumatic OA. Future studies to determine the efficacy of procyanidins on disease progression at different stages are needed, and are currently under investigation. Upon further validation and development, procyanidins may become an attractive therapeutic strategy in both OA prevention and treatment.

Since current nutraceutical products may have an overall dose of 50 mg/kg daily, the dose of procyanidins used in this study may be considered on the high side of the effective dosage range. Our study indicates that procyanidins B2 and B3 are at least a part of the active ingredients of procyanidins. Upon further validation, a procyanidins product with enriched active ingredients, such as procyanidins B2 and B3, could be developed, and may be effective at a significantly reduced dosage. Furthermore, integration of procyanidins with other nutraceuticals that have shown OA-disease and symptom modifying effects, such as curcumin [16] and green tea polyphenol epigallocatechin gallate (EGCG) [17], could provide an enhanced effect in mitigating OA pathogenesis. Such approaches will also provide a basis for further research and development for the pharmaceutical intervention for treating OA.

As OA is a disease of the entire joint organ [3], alteration of VEGF expression may not only affect articular cartilage, but also the underlying bone and surrounding synovium in OA disease pathogenesis [54]. While VEGF aids in bone repair under physiological conditions, in the OA joint, aberrant VEGF signaling promotes endochondral ossification, disrupting homeostasis in the calcified cartilage layer below, as well as subchondral bone remodeling, potentially leading to thickening of the subchondral bone layer, or introduction of osteophytes [54]. In future studies, we will evaluate whether procyanidins preserve the integrity of calcified cartilage as endochondral ossification progresses, and determine whether procyanidins prevent such alterations in the subchondral bone.

## 4. Materials and Methods

### 4.1. Primary Human Chondrocytes Culture

All human studies were approved by the Albert Einstein College of Medicine Institutional Review Board. Chondrocytes were isolated from human patients who underwent knee replacement surgery (*n* = 4, females, ages 49–64), as described previously [16,82]. Chondrocytes were cultured in Dulbecco’s Modified Eagle Medium: Nutrient Mixture F-12 (DMEM/F12) supplemented with 10% fetal bovine serum (FBS), at 37 °C, 5% CO_2_. All experiments were carried out at the 2nd passage.

### 4.2. Procyanidins Preparation and Treatment In Vitro

Procyanidins (Pine bark extract, Natrol, Chatsworth, CA, USA) were dissolved in water (MilliQ, EMD Millipore, Billerica, MA, USA) and then centrifuged at 2000× *g* for 5 min to remove insoluble components. The solution of procyanidins was collected, reconstituted at 20 mg/mL in phosphate-buffered saline (PBS) and filtered through a 0.22 µm syringe filter as the stock solution.

The chondrocytes were incubated with 10 ng/mL human recombinant IL-1β (Gemini Bio-Products, Broderick, CA, USA) for 30 min prior to the treatment with procyanidins. Cells were then incubated for 24 h with different concentrations of procyanidins specifically indicated in the relevant figures in Results.

To examine the effect of procyanidins on VEGF signaling, human primary chondrocytes were incubated with 0–100 µg/mL procyanidins [29,32,83] at 37 °C, 5% CO_2_ for 1 h. Then the cells were stimulated with 100 ng/mL exogenous human recombinant VEGF (Gemini) for 5 min. The cells were then lysed for Western blot to detect the expression of total VEGFR-2 and the phosphorylation of VEGFR-2.

In order to examine whether procyanidin B2, B3 individually, or the combination of B2 and B3 have similar effects as the procyanidins mixture, human primary chondrocytes were divided into four groups. Each group of cells was incubated with 1 µg/mL of procyanidin B2 (Sigma-Aldrich, St. Louis, MO, USA), 1.25 µg/mL of procyanidin B3 (AdooQ BioSciences, Irvine, CA, USA), a combination of 1 µg/mL of procyanidin B2 and 1.25 µg/mL of procyanidin B3, or 50 µg/mL of procyanidins prepared as described above, in the presence of 100 ng/mL human recombinant VEGF for 24 h. The dosages of procyanidin B2 and B3 were determined by the percentage of B2 and B3 in pine bark extract from previously published HPLC data [28]. Cells were lysed after treatments and processed for real-time PCR or Western Blot.

### 4.3. DMM Mouse Model and Procyanidins Treatment In Vivo

All animal studies were approved by the Albert Einstein College of Medicine Institutional Animal Care and Use Committee. DMM was established in adult C57BL/6 mice (males, 5–6 months of age) by surgically transecting the medial meniscotibial ligament (MMTL) in the right hind limb knee joint [16]. Briefly, an incision was made at medial to the patellar tendon to provide access to the articular joint. The patellar tendon was moved slightly to allow blunt dissection of the fat pad, providing visualization of the MMTL. The MMTL of the medial meniscus was then transected, creating destabilization in the right knee joint. The medial meniscus, though destabilized, was not removed. The joint capsule and skin were then closed with sutures.

Immediately after the DMM surgery, 50 mg/kg of procyanidins or phosphate-buffered saline (PBS) as vehicle control were administered via oral gavage once daily for 8 weeks. At 8 weeks post-surgery, groups of procyanidin-treated and vehicle-treated animals (*n* = 4/group) were euthanized.

### 4.4. Safranin-O and Immunohistochemistry

The right, hind limbs of the mice were fixed in 10% zinc formalin for 24 h, decalcified in formical-2000 for 24 h, embedded in paraffin and sectioned. Sections were deparaffinized with xylene and ethylene glycol monomethyl ether (EGME). Safranin-O fast green staining was used to visualize proteoglycans in the articular cartilage. OA severity was evaluated using the OARSI scoring system [20].

Sections for immunohistochemistry were incubated 16–18 h at 4 °C with antibodies against cleaved aggrecan (NITEGE; IBEX Technologies, Montreal, QC, Canada) and cleaved type II collagen (Col2 3/4C short; IBEX Technologies), MMP-13 (Abcam, Cambridge, MA, USA) and ADAMTS5 (Abcam), and VEGF (Abcam) followed by 25-min incubation with anti-rabbit secondary antibody (Biocare Medical, Concord, CA, USA) and visualization with 3,3′-diaminobenzidine chromogen (Vector Laboratories, Burlingame, CA, USA) for 3–5 min. Immunohistochemistry staining with rabbit immunoglobulin G (IgG) in serial tissue sections served as an isotype control.

To evaluate intensity of cleaved type II collagen or cleaved aggrecan cleavage epitopes, the reciprocal intensity of the immunostaining in the cartilage matrix was quantified. The light intensity value was measured using color picker in Adobe Photoshop (Adobe Systems, San Jose, CA, USA) [21]. These values were measured from six random locations of the joint, three from each the femoral condyles and tibial plateau in the direction from the posterior to the anterior of the joint, and were then averaged. Percentages of positive MMP-13, ADAMTS5 and VEGF chondrocytes were determined by counting the number of immunostained cells and dividing by the total number of chondrocytes visualized by using a hematoxylin counterstain (Vector Laboratories). ImageJ (National Institutes of Health, Bethesda, MD, USA) was used to count the number of stained cells.

### 4.5. RNA Isolation and Real-Time PCR

Total RNA from human chondrocytes was isolated with a GeneJet RNA Purification Kit (ThermoFisher Scientific, Waltham, MA, USA). Mouse cartilage samples were carefully harvested from 10 µm-thick sections and total RNA was isolated by using a PureLink FFPE (formalin-fixed, paraffin-embedded) RNA Isolation Kit (ThermoFisher Scientific). First strand complementary DNA was synthesized by using an iScript reverse transcriptase kit (Bio-Rad Laboratories, Hercules, CA, USA). Real-time PCR was performed to determine relative gene expression by using SYBR green supermix (Bio-Rad Laboratories) and gene specific primers (Table 2). Glyceraldehyde 3-phosphate dehydrogenase (GAPDH) was used as a housekeeping control.

### 4.6. Protein Extraction and Western Blot

Human cartilage specimens were cut into ~25 mm^3^ pieces and washed with ice cold PBS 3 times, then snap frozen in liquid nitrogen. One piece of each specimen was ground with a dismembrator (Sartorius, Göttingen, Germany). To extract protein from the tissue, 100 µL Mammalian Protein Extraction Reagent (M-PER; ThermoFisher Scientific) containing protease inhibitor cocktail (Roche, Basel, Switzerland) was added to the ground tissue. After three cycles of freeze and thaw, total protein contained in the supernatants was obtained by centrifuge at 12,000× *g* at 4 °C for 15 min. Western blots were performed as previously described [82]. Briefly, approximately 10 µg of protein (from cartilage extracts or cell lysate) was separated by sodium dodecyl sulfate-polyacrylamide (SDS-PAGE) gel electrophoresis in a 4%–20% gradient polyacrymide gel (Bio-Rad Laboratories), and transferred to a polyvinalidene diflouride membrane (PVDF) membrane (Bio-Rad Laboratories). The membranes were incubated with primary antibodies against VEGFR-2, or phosphorylated VEGFR-2 (both from Cell Signaling Technology, Danvers, MA, USA), followed by incubation with horseradish peroxide conjugated secondary antibody. Enhanced chemiluminescence (ECL) kit (Buckinghamshire, UK) was used to detect the binding of antibodies to target proteins. An antibody against β-actin was used as loading control. ImageJ software was used to quantify the intensities of the Western blot bands.

### 4.7. Statistical Analysis

The results are expressed as mean + SD. Significance was determined using Student’s *t*-test in Microsoft Excel, or with a normalcy test followed by one-way ANOVA with Tukey post-hoc test as appropriate (GraphPad). *p* < 0.05 was considered as statistically significant.

## 5. Conclusions

The current study was aimed at proving the concept that procyanidins exert a therapeutic effect in mitigating OA, and identifying its possible pathway targets and potential active ingredients. We provide direct evidence that daily oral administration of procyanidins mitigate OA pathogenesis in mice subjected to DMM surgery. Our studies further demonstrate that the chondroprotective effects of procyanidins may be due to, at least in part, suppressing VEGF-mediated signaling in OA chondrocytes. Further studies on procyanidin-based therapeutics are warranted to confirm this new possible therapeutic approach in mitigating OA pathogenesis.

## Figures and Tables

**Table 1:** Composition of procyanidins and their therapeutic effects or potential effects.

Known Procyanidins	Therapeutic Effects or Potential Effects
**A-Type Dimers**	
Procyanidin A1	immune modulation [33,34], cholesterol modulation [35], anti-oxidative effects [20]
Procyanidin A2	anti-inflammation [36], anti-diabetic effect [37]
**B-Type Dimers (of interest in pine bark)**	
Procyanidin B1	anti-inflammation [38], anti-hepatitis-C [39], anti-cancer [40], anti-oxidative effects [19]
Procyanidin B2	anti-diabetic effects [41,42], anti-inflammation [43], anti-cancer [44], anti-oxidative effects [19]
Procyanidin B3	arthritis modulation [32], anti-cancer [22], anti-inflammation [45], anti-oxidative effects [20]
Procyanidin B4	anti-oxidative effects [46]
Procyanidin B5	anti-cancer [28]
Procyanidin B6	
Procyanidin B7	
Procyanidin B8	
**Trimeric B-type**	
Procyanidin C1	immune modulation [47,48], cardiovascular-protective effects [49], anti-inflammation [50], anti-cancer [51]
Procyanidin C2	

**Table 2:** Primer sequences used for real-time PCR.

Gene in Mice	Sequence of Selected Primer Pairs	Gene in Humans	Sequence of Selected Primer Pairs
*Vegf*	F: GCAGACTATTCAGCGGACTCA	*VEGF*	F: AGGGCAGAATCATCACGAAGT
	R: GGGAGTGAAGAACCAACCTCC		R: AGGGTCTCGATTGGATGGCA
*MMP-1*	F: CCTCGTTGGACCAAAACACA	*ADAMTS-5*	F: GAACATCGACCAACTCTACTCCG
	R: GCGATGGCATCTTCCACAA		R: CAATGCCCACCGAACCATCT
*MMP-3*	F: TCCTGATGTTGGTGGCTTCA	*IL-1β*	F: CTCCGGGACTCACAGCAAAA
	R: CACACTCTGTCTTGGCAAATCC		R: GCCCAAGGCCACAGGTATT
*MMP-13*	F: TCACCTGATTCTTGCGTGCTA	*IL-6*	F: CCTGAACCTTCCAAAGATGGC
	R: CAGATGGACCCCATGTTTGC		R: TTCACCAGGCAAGTCTCCTCA
*Adamts-5*	F: GCTGCTGGTAGCATCGTTACTG	*RANKL*	F: CAACATATCGTTGGATCACAGCA
	R: GAGTGTAGCGCGCATGCTT		R: GACAGACTCACTTTATGGGAACC
*Il-1β*	F: GCTTCCTTGTGCAAGTGTCTGA	*HIF-1α*	F: GAACGTCGAAAAGAAAAGTCTCG
	R: TCAAAAGGTGGCATTTCACAGT		R: CCTTATCAAGATGCGAACTCACA
*Tnf-α*	F: AGGGATGAGAAGTTCCCAAATG	*ACAN*	F: GTGCCTATCAGGA
	R: GGCTTGTCACTCGAATTTTGAGA		R: GATGCCTTTCACCACGACTTC
*Il-6*	F: TCGGAGGCTTAATTACACATGTTC	*MMP-1*	F: TCCACAAATGGTGGGTACAA
	R: TGCCATTGCACAACTCTTTTCT		R: GGTGACACCAGTGACTGCAC
*Rankl*	F: AGGCTCATGGTTGGATGTGG	*MMP-13*	F: ACTGAGAGGCTCCGAGAAATG
	R: GCATTGATGGTGAGGTGTGC		R: GAACCCCGCATCTTGGCTT
*Hif-1α*	F: GGATGAGTTCTGAACGTCGAAAAG	*TNF-α*	F: CGAACATCCAACCTTCCCAAAC
	R: GTGGCAACTGATGAGCAAGC		R: TGGTGGTCTTGTTGCTTAAAGTTC
*Col2a1*	F: AGCAGGAATTCGGTGTGGAC	*MMP-3*	F: TCACAGTTGGAGTTTGACCC
	R: GACATCAGGTCAGGTCAGCC		R: AAGTGCCCATATTGTGCCTTC
*Acan*	F: TGGGATCTACCGCTGTGAAGT	*COL2a1*	F: AGCAGGAATTCGGTGTGGAC
	R: CTCGTCCTTGTCACCATAGCAA		R: GACATCAGGTCAGGTCAGCC
*Glyceraldehyde 3-phosphate dehydrogenase (Gapdh)*	F: AGGTCGGTGTGAACGGATTTG	*GAPDH*	F: CATGGAGAAGGCTGGGGCTCATTTG
	R: TGTAGACCATGTAGTTGAGGTCA		R: GGGGTGCTAAGCAGTTGGTGGT

## Abbreviations

ACAN	aggrecan
ADAMTS5	A disintegrin and metalloproteinase with thrombospondin motifs 5
Col2a1	type II collagen
DMM	destabilization of the medial meniscus
ECM	extracellular matrix
GRAS	generally recognized as safe
Hif1-α	hypoxia-inducible factor 1-alpha
IL	interleukin
NSAIDs	nonsteroidal anti-inflammatory drugs
MMP	matrix metalloproteinase
OA	osteoarthritis
PC	procyanidins
PCR	polymerase chain reaction
RANKL	receptor activator of nuclear factor κ-B ligand
ROS	reactive oxygen species
TNF-α	tumor necrosis factor α
VEGF	vascular endothelial growth factor
VEGFR-2	vascular endothelial growth factor receptor 2
